# Restitution characteristics of His bundle and working myocardium in isolated rabbit hearts

**DOI:** 10.1371/journal.pone.0186880

**Published:** 2017-10-26

**Authors:** Shangwei Huang, Liqun Wu, Jian Huang, Nuttanont Panitchob, Nan Hu, Ravi Ranjan, Derek J. Dosdall

**Affiliations:** 1 Department of Cardiology, Shanghai Ruijin Hospital, Shanghai Jiao Tong University School of Medicine, Shanghai,China; 2 Department of Internal Medicine, Division of Cardiology, University of Utah, Salt Lake City, UT, United States of America; 3 Department of Medicine, University of Alabama at Birmingham, Birmingham, AL, United States of America; 4 The Nora A. Eccles Cardiovascular Research and Training Institute, University of Utah, Salt Lake City, UT, United States of America; 5 Department of Internal Medicine, Division of Epidemiology, University of Utah, Salt Lake City, UT, United States of America; 6 Department of Surgery, Division of Cardiothoracic Surgery, University of Utah, Salt Lake City, UT, United States of America; University of Minnesota, UNITED STATES

## Abstract

The Purkinje system (PS) and the His bundle have been recently implicated as an important driver of the rapid activation rate after 1–2 minutes of ventricular fibrillation (VF). It is unknown whether activations during VF propagate through the His-Purkinje system to other portions of the the working myocardium (WM). Little is known about restitution characteristic differences between the His bundle and working myocardium at short cycle lengths. In this study, rabbit hearts (n = 9) were isolated, Langendorff-perfused, and electromechanically uncoupled with blebbistatin (10 μM). Pacing pulses were delivered directly to the His bundle. By using standard glass microelectrodes, action potentials duration (APD) from the His bundle and WM were obtained simultaneously over a wide range of stimulation cycle lengths (CL). The global F-test indicated that the two restitution curves of the His bundle and the WM are statistically significantly different (P<0.05). Also, the APD of the His bundle was significantly shorter than that of WM throughout the whole pacing course (P<0.001). The CL at which alternans developed in the His bundle vs. the WM were shorter for the His bundle (134.2±13.1ms vs. 148.3±13.3ms, P<0.01) and 2:1 block developed at a shorter CL in the His bundle than in WM (130.0±10.0 vs. 145.6±14.2ms, P<0.01). The His bundle APD was significantly shorter than that of WM under both slow and rapid pacing rates, which suggest that there may be an excitable gap during VF and that the His bundle may conduct wavefronts from one bundle branch to the other at short cycle lengths and during VF.

## Introduction

Ventricular fibrillation (VF) is an important cause of sudden cardiac death. Recent studies have shown that the Purkinje system plays an active role in VF onset and maintenance.[1–3] However, little is known about the role of the proximal ventricular conduction system (i.e., His bundle, and bundle branches).

Published studies have demonstrated that the Purkinje system (PS) is an important driver of VF activation in the maintenance of long duration VF (LDVF)(VF>2 min).[1–3] PS activity was present in 84% of fibrillatory wave fronts during induced VF in dog isolated hearts and was responsible for driving the rapid activation rate during LDVF.[3] Also, the PS has the same or shorter action potential duration (APD) and refractory periods as cardiomyocytes under rapid pacing rates[4], which suggests that the PS may experience periods of time when it is excitable between activations even at the rapid activation rates that occur during VF.

A recently published study has shown that during prolonged VF the His bundle exhibits similar activation patterns as the PS.[5] This suggests that the His bundle and the PS remain electrically linked during VF and that pacing and capture of the His bundle may lead to capture of the PS during VF. However it is unclear whether the APD and refractory periods of the His bundle have the similar electrophysiological properties with the Purkinje fibers. In this study, we attempted to further characterize the action potential characteristics of the His bundle compared to the underlying working myocardium at short cycle lengths. This will help identify the potential role of the conduction system in rapid ventricular arrhythmias such as ventricular tachycardia and VF.

## Materials and methods

All procedures involving animals were performed in accordance with the Guide for the Care and Use of Laboratory Animals[6], and the protocol was approved by the Institutional Animal Care and Use Committee of the University of Utah. All efforts were made to minimize suffering.

### Animal preparation

New Zealand white rabbits (n = 9, 2–4 kg, Western Oregon Rabbit Company, Philomath, Oregon, USA) of either sex were anesthetized using intramuscular injections of 30 mg/kg ketamine and 5 mg/kg xylazine, followed by intravenous injections of 10 mg/kg ketamine, 3 mg/kg xylazine and 500 IU of heparin. After a median sternotomy, the hearts were excised and isolated rapidly in 4°C Tyrode’s solution, and then were Langendorff-perfused with 37°C Tyrode’s solution. The perfusion pressure was maintained at 60 to 70 mmHg. The hearts were also superfused by warm Tyrode’s solution, with temperature maintained at 37±0.5°C. The composition of Tyrode’s solution (in mM) was 130 NaCl, 1.2 NaH_2_PO_4_, 1 MgCl_2_, 4 KCl, 1.8 CaCl_2_, 24 NaHCO_3_, 11.2 Glucose, and 0.04 g/L bovine albumin and it was oxygenated with O_2_ and CO_2_ to maintain a pH of 7.4±0.05. The excitation-contraction uncoupler blebbistatin (10 mM/L; Calbiochem®, EMD Biosciences, Inc. LA Jolla, CA) was added to Tyrode’s solution to suppress motion artifacts in the recordings.[7]

### Signal recordings

**Fig 1:**
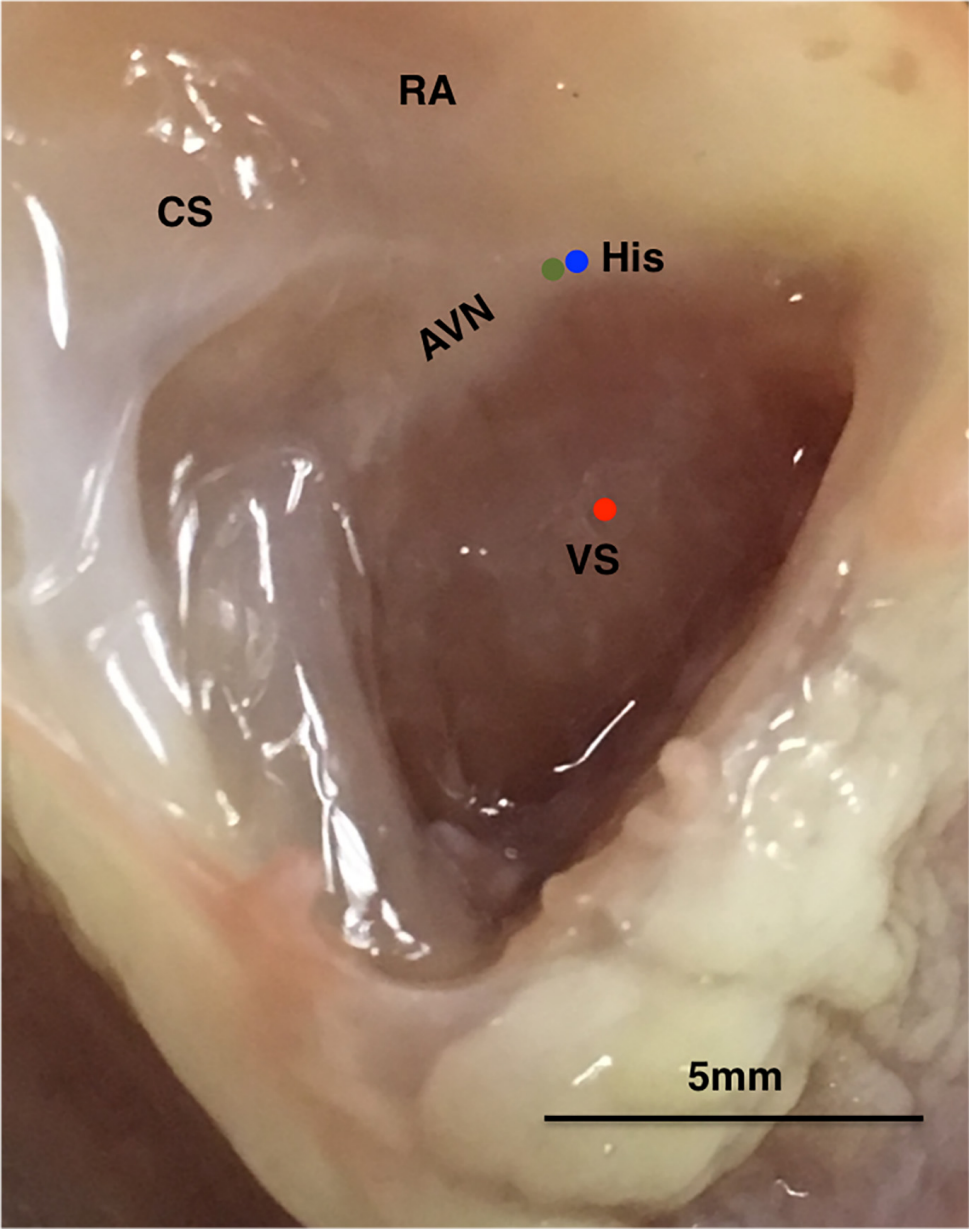
Photograph of the experimental rabbit heart showing anatomical landmarks and impalement sites. CS, coronary sinus; AVN, atrioventricular node; His, His bundle; RA, right atrium; VS, ventricular septum. Green dot indicates the recording site of the bipolar electrode. Action potentials were recorded from the His bundle (blue dot) and endocardium (red dot), respectively.

**Fig 2:**
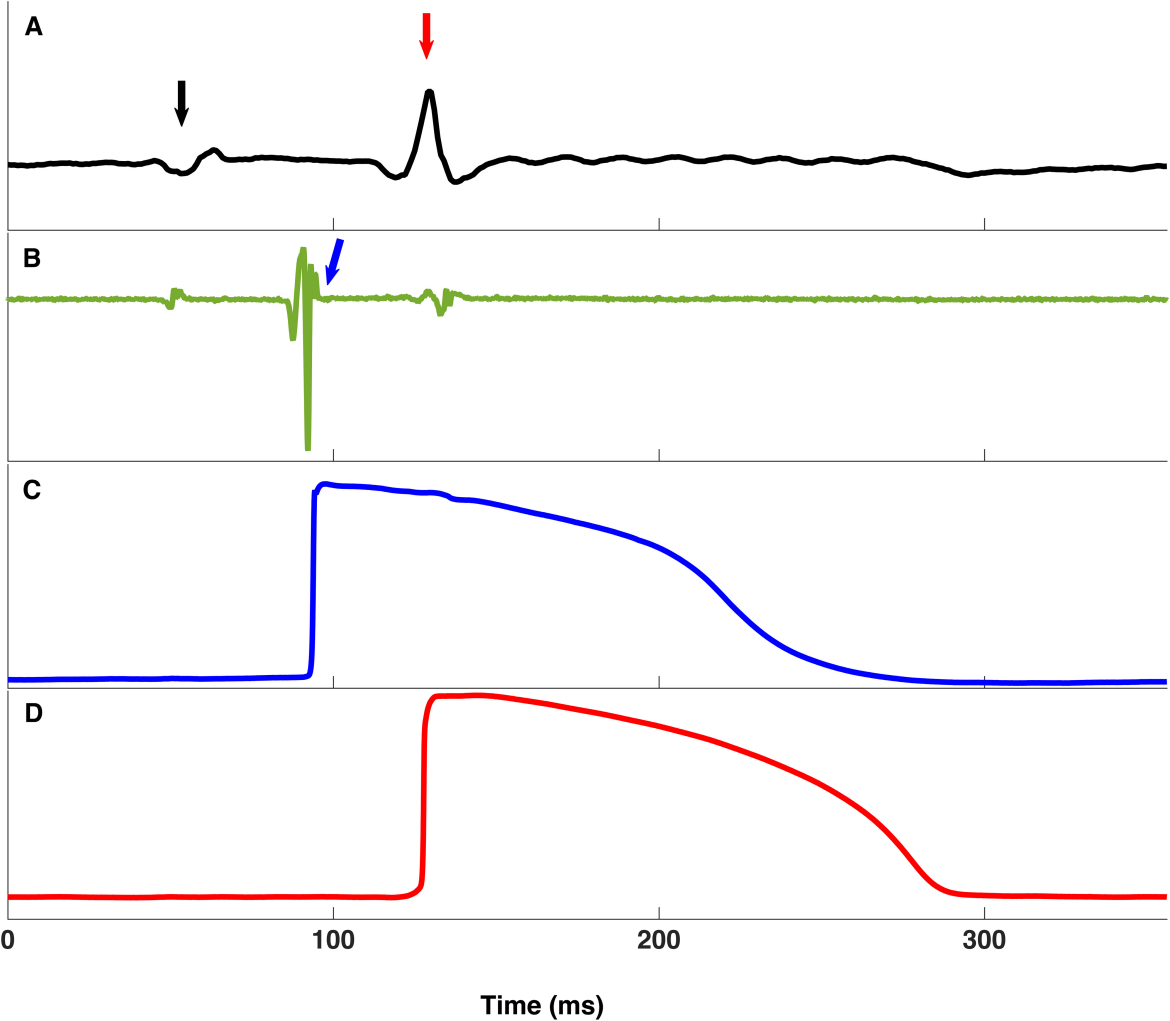
**Example of the pseudo ECG (A), bipolar electrogram (B) and microelectrode recordings of the His bundle (C) and adjacent working myocardium (D).** In the pseudo ECG panel, the first signal (black arrowed) is P wave and the second one (red arrow) is QRS wave. Panel B is the derivatives of the bipolar electrogram signals. The first and third deflections in panel B are temporally aligned with the P and QRS wave in panel A and the second deflection (green arrow) is the His bundle signal.

The right atrium was removed to expose the basal right ventricular septum and the region containing the His bundle, as shown in Fig 1. A bipolar electrode was placed on the His bundle for signal discrimination and pacing. Two standard glass microelectrodes (tip resistance, 1 to 5 MΩ, filled with 3 mol/L KCl) were used to record the intracellular action potential (AP) from the His and the right ventricular septal endocardium simultaneously. One microelectrode impaled the His bundle within 2 mm of the bipolar electrode, which was used to verify that the action potential (AP) aligned with the His bundle signal in the bipolar electrode. The other microelectrode recorded the WM signal on the endocardium within 10 mm of the His bundle impalement site. An Ag-AgCl reference electrode for the intracellular recording electrode was placed in the perfusate. APs were recorded with DC coupling as the difference in voltage between the intracellular microelectrode and the extracellular Ag-AgCl reference electrode. The signals were acquired by an Axoclamp 900A amplifier (Axon Instruments, USA). Also, another three electrodes were placed in the perfusate to calculate pseudo-ECG. All the signals were recorded and monitored in real time using LabChart® software through a Powerlab 16/30 system (AD Instruments, Colorado Springs, CO, USA) (Fig 2).

### Experimental protocol

**Fig 3:**
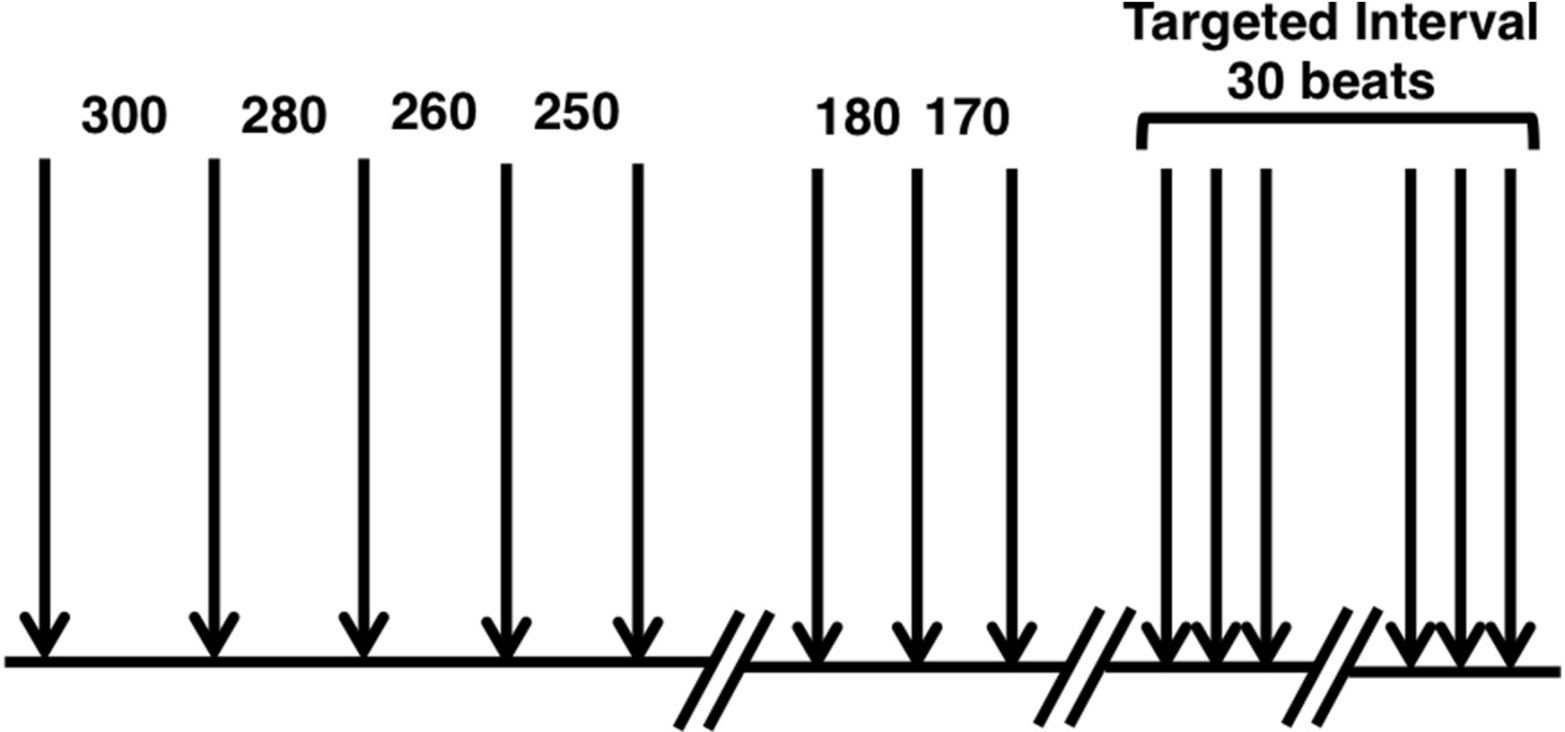
Decremented-pacing protocol for measuring restitution properties. The initial interval was 300ms and was decremented to 260ms by 20-ms steps. Below 260 ms, it was reduced in 10-ms steps to the target interval, and thereafter kept at the target interval for 30 beats. Each cycle length was in ms.

Electrical pulses of 0.2 ms duration and twice diastolic threshold were delivered through the bipolar electrode placed on the His bundle. A steady-state pacing protocol used as in a previously published study[8] was used to determine the restitution characteristics. The S1–S1 interval was initially 300ms and was progressively decreased to 260ms by 20-ms steps, and then was reduced in 10-ms steps to the target interval, and thereafter kept at the target interval for 30 beats (Fig 3). After finishing the restitution curve, the same protocol was used to pace from the ventricular apex to determine if capture of the WM was lost at the same cycle length (CL) with the one paced from the His bundle.

### Statistical analysis

The data were selected if there was a stable microelectrode recording and the last 10 beats of the 30 beats at each pacing CL were analyzed. The APD was measured using LabChart^®^ software at 90% repolarization (APD_90_). The diastolic interval (DI) was defined as the interval from the end of the repolarization time of the previous beat to the activation time of the next beat. The confirmative analysis focused on comparing if the relationship between APD and DI are the same for the His bundle and working myocardium. We assume the non-linear relationship between APD and DI (ms) as follows.

$$y=\alpha -\beta e^{-x/\tau }$$ (1)

where y denotes the value of APD and x for the value of DI, and α, β and τ are parameter coefficients being estimated from the data. We first assumed that both the His bundle and WM shared the same parameters for α, β and τ in the above nonlinear model (1) and fit a common-parameter model. Then, we fit the non-linear model assuming that each of the parameters, α, β and τ, was different between the His bundle and the WM group. A global F-test was used to examine if two models (the model assuming common parameters and the model assuming different parameters) were statistically significantly different from each other. If the F-test indicates that the two models were significantly different, we can conclude that the relationship between APD and DI (or between APD and CL) was significantly different across the two (His bundle and WM) groups. We then fit the non-linear model (1) again and re-parametrized the model to test if any of the three parameters (α, β and τ) were significantly different between the His and WM group. If any of the parameters was found significantly different between the His bundle and WM group, we estimated a separate coefficient for that parameter for the His bundle and the WM group. Otherwise, if a parameter was found to be not significantly different between the His bundle and WM group, we used the same coefficient for that parameter for both groups. Our final model includes the common parameter(s) and the separate parameter(s) based on the above processes. If the global F-test indicated that the difference between the common parameters model and the separate parameters model was not significantly different from each other, we concluded that the relationship between APD and DI were the same for the two groups, and the common parameter model was used as the final model.

For the relationship between APD and CL, either linear or non-linear regression analyses have been performed in previous studies. Hence, we used a derived outcome, the area under the curve (AUC), for comparing between groups. The AUC was calculated using the two-way curve with APD on the y-axis and CL on the x-axis with the same range of CL for all animals within the His bundle and WM groups. The AUC was calculated by summing up the areas of trapezoids generated by projecting each data point of APD and CL pair onto the x-axis (a triangular shape was obtained for the area from point (0,0) to the pair of APD and the minimal CL). Although AUCs are continuous measurements, due to fact that the AUCs are not normally distributed for both WM and the His bundle groups a Wilcoxon signed rank test was used for comparing AUCs between the His bundle and WM groups.

All statistical analyses were performed using SAS (SAS Inc., Cary, NC, USA) version 9.4. Test results with p-values <0.05 were considered statistically significant.

## Results

### APD restitution curves of the His bundle and working myocardium

**Fig 4:**
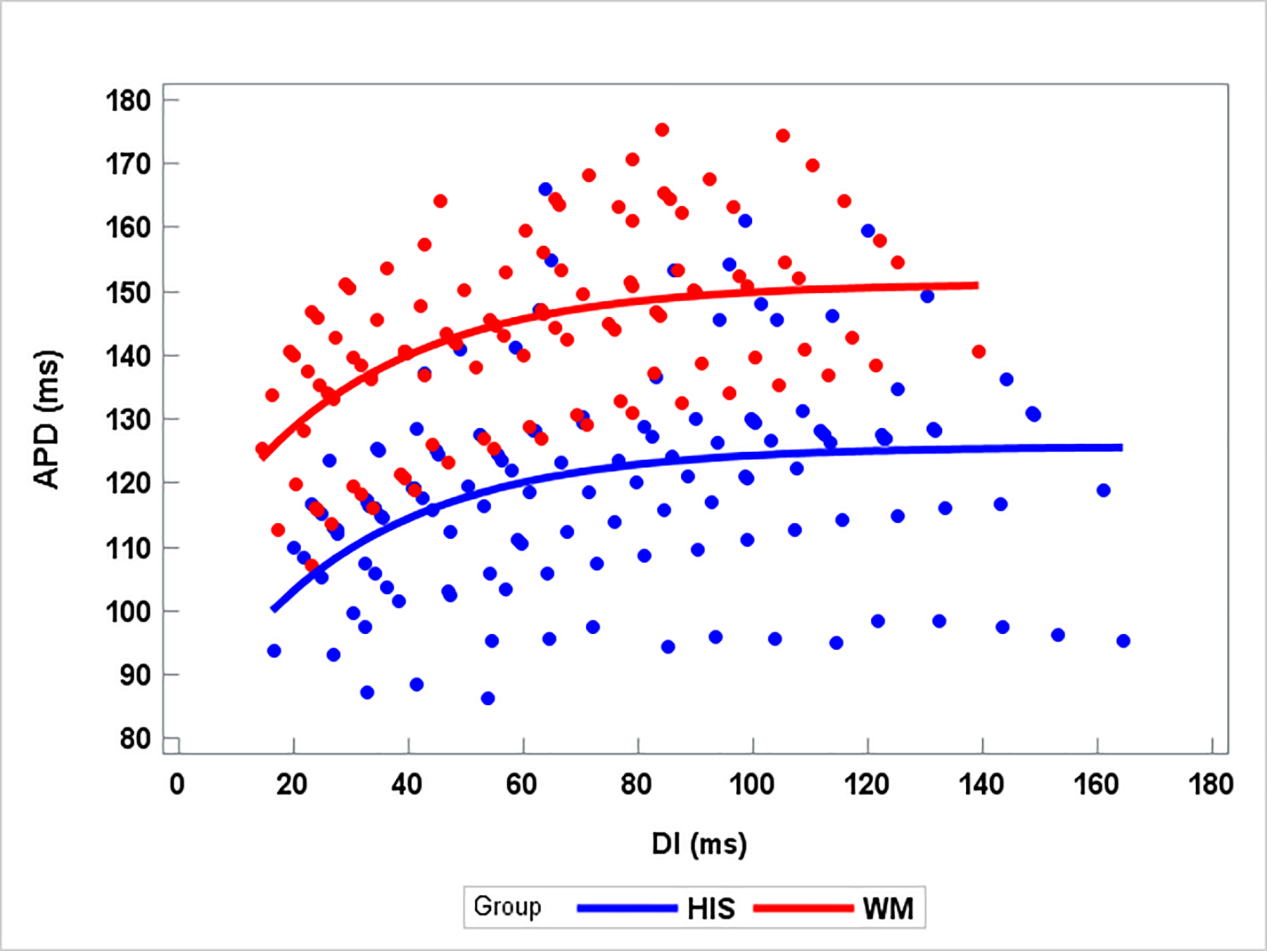
Scatterplot of APD versus DI with fitted lines. The blue dots and line denote the data points and the fitted line for the His bundle group, and the red dots and lines for the WM group.

Fig 4 presents the scatterplot of APD versus DI for both the His bundle and WM groups with the fitted restitution curves. Both common-parameter and separate-parameter models were fitted for APD versus DI between the His bundle and WM groups. The global F-test indicated that the two models are statistically significantly different (p<0.000001). By examining each of the three parameters in the non-linear model, we found that only the α parameter in Eq (1) is significantly different between the two groups. The expected difference in α between the two groups is 31.21 (95% CI: 15.33 to 47.09, p<0.0001), with the WM groups having a larger value. Thus, we conclude that the relationship between APD and DI are different across the His bundle and WM groups.

#### Rate dependence of APD in His bundle and working myocardium

**Fig 5:**
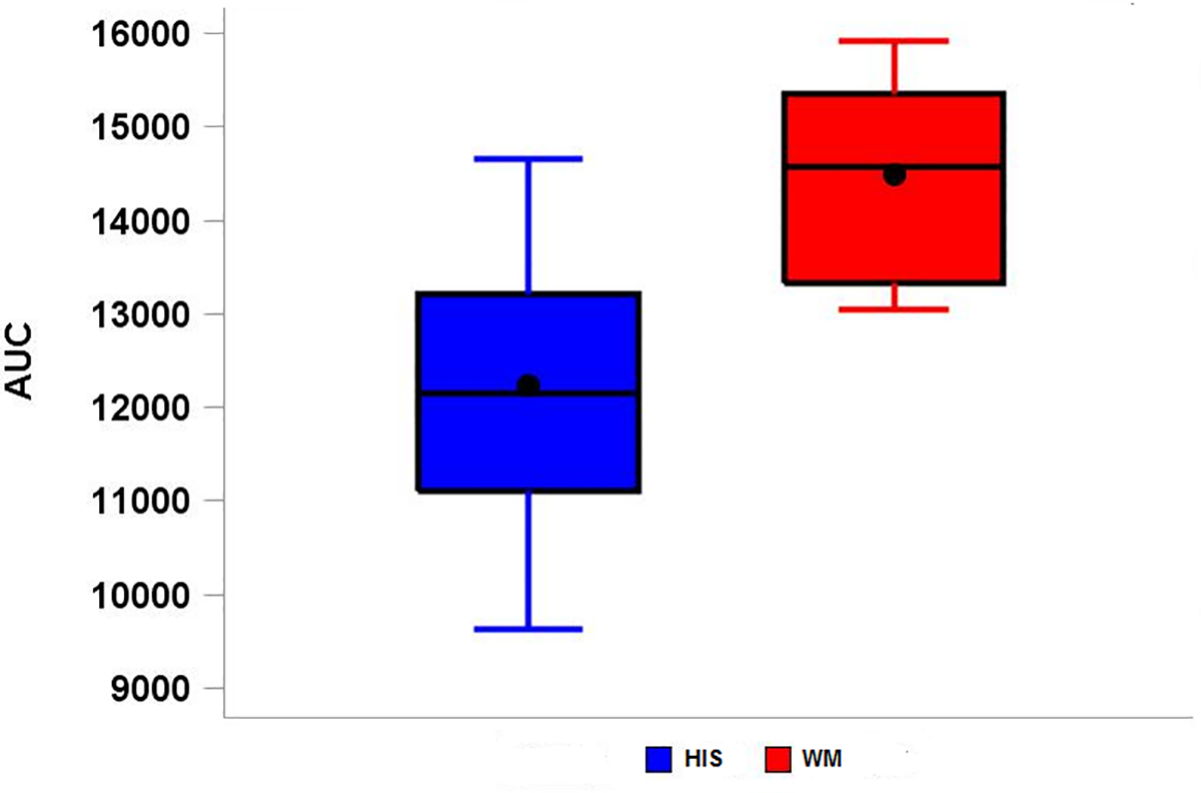
Boxplot of AUC. The blue boxplot is for the His bundle group, and the red one is for the WM group.

We performed the Wilcoxon signed rank test to compare the paired AUCs between the His bundle and WM group under the null hypothesis that the AUCs under both groups are equal. At the 0.05 test level, this null hypothesis was rejected (p = 0.018) and we concluded that the WM and the His bundle groups have statistically significantly different AUCs that describe the relationship between APD and CL (Fig 5).

#### APD alternans and 2:1 block in His bundle and working myocardium

**Fig 6:**
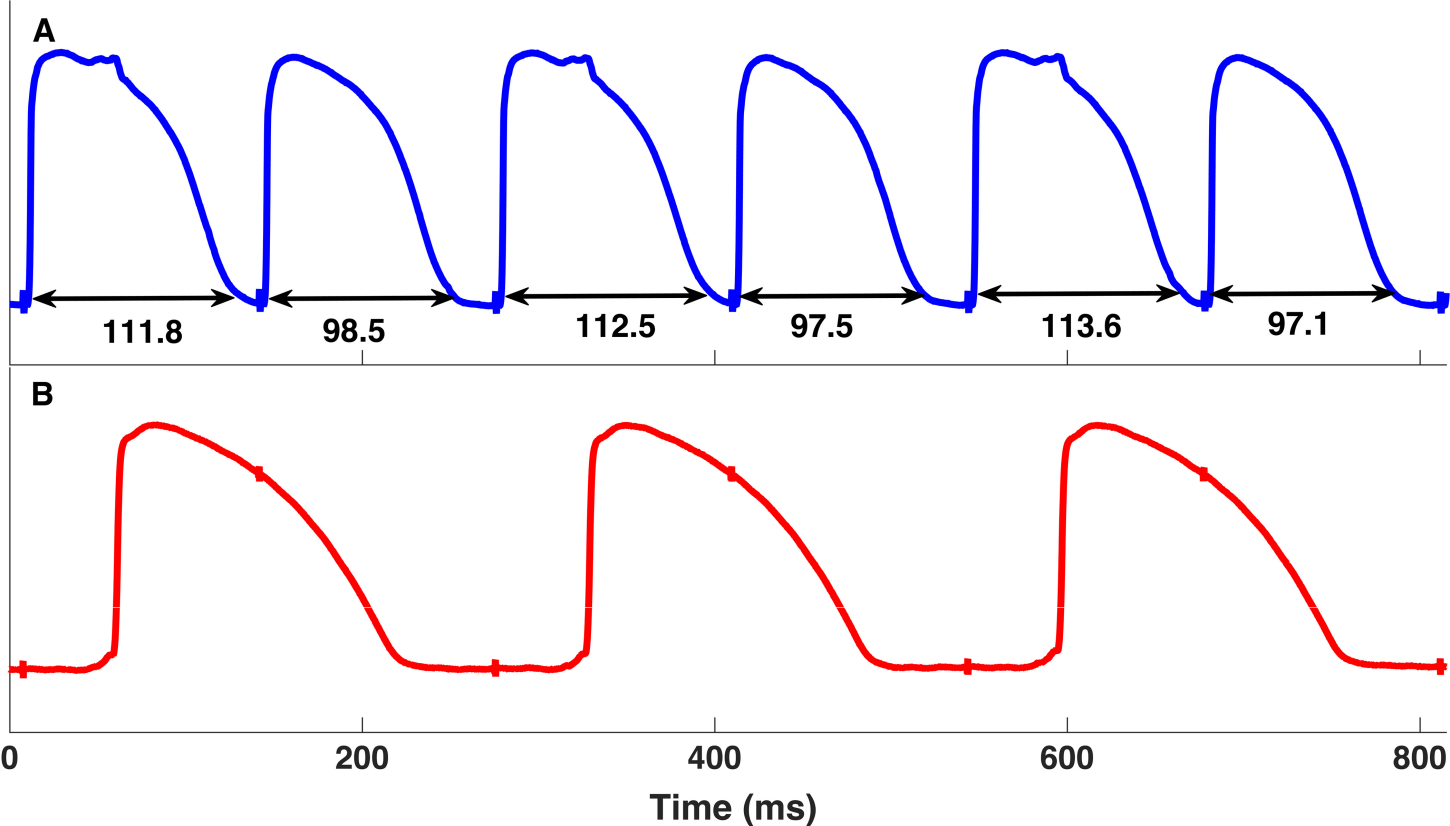
**Action potential duration alternans continuously recorded in the His bundle (A) and 2:1 block in the adjacent working myocardium (B) at the cycle length of 130ms pacing from the His bundle.** The spikes in the recordings are the pacing artifacts. In panel A, the APD_90_ of the His bundle alternated in a long-short pattern. The long APDs ranged from 111.8ms to 113.6ms, while the short ones ranged from 97.1ms to 98.5ms. The working myocardium lost one to one capture when the His bundle had alternans.

**Fig 7:**
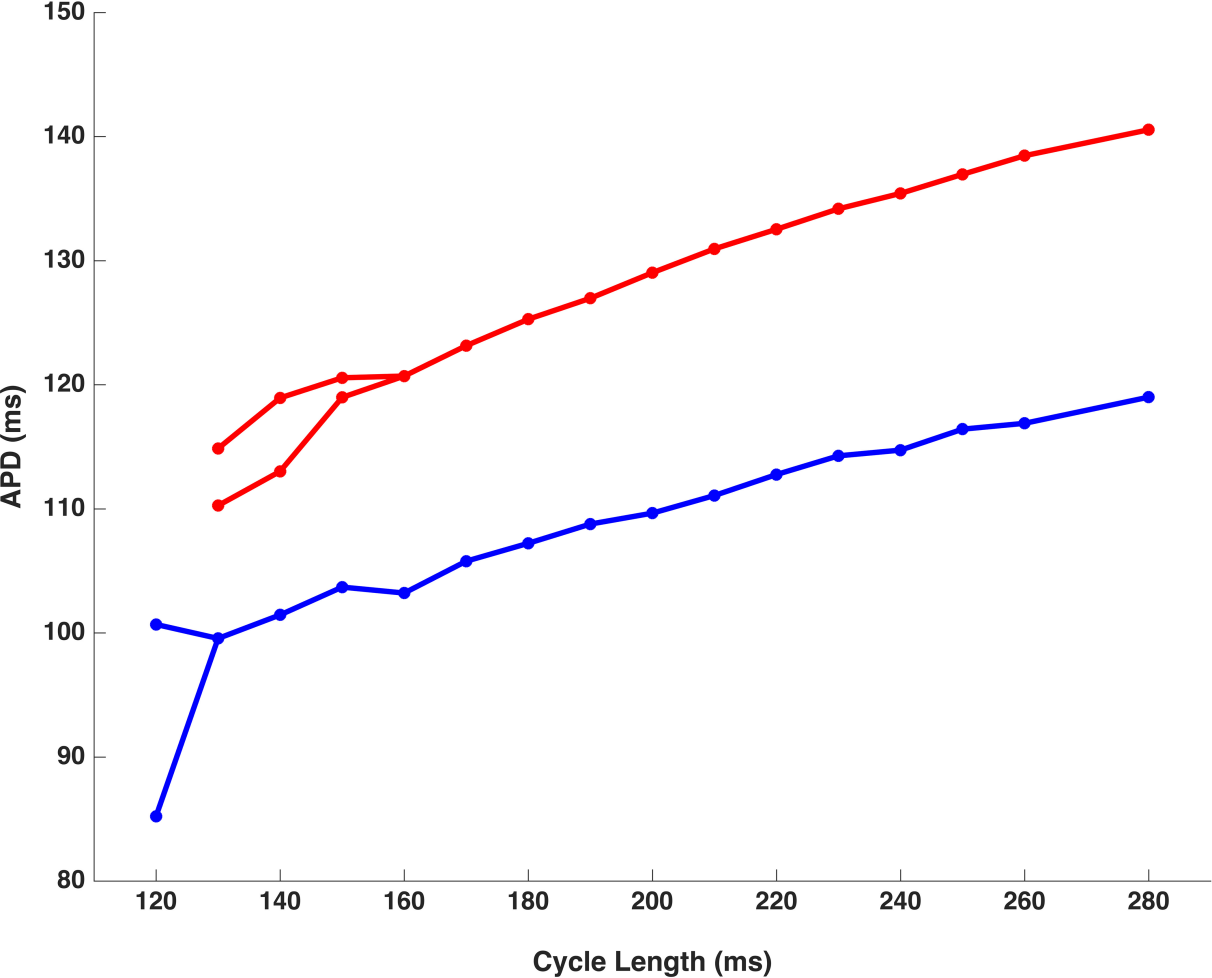
Action potential durations (APD_90_) during dynamic pacing in the His bundle (blue dots and curves) and working myocardium (red dots and curves). As cycle length progressively shortened, a transition from 1:1 capture to 2:1 block with alternation between long and short action potentials occurred.

During dynamic pacing, as the target interval closed to the CL where the His bundle or WM developed 2:1 block, the onset of APD alternans usually occurred. The His alternans developed in 7 of the 9 animals (77.8%). Among these 7 animals that had His alternans, 4 rabbits also displayed WM alternans. There was another 1 animal (11.1%) that had only WM alternans with no His bundle alternans. The His bundle alternans developed at an average CL of 134.2±13.1ms. Compared with the His bundle alternans, the WM alternans happened at longer CL (148.3±13.3ms, p<0.05). Similar to alternans, 2:1 block also developed at shorter CLs in the His bundle than in WM (130.0±10.0 vs. 145.6±14.2ms, P<0.01). Furthermore, we found that most alternans of the His bundle occurred when the WM had 2:1 block (9 out of 12, 75.0%). Figs 6 and 7 showed the APD_90_ during dynamic pacing in the His bundle and WM from one animal. The WM had alternans and 2:1 block at 150ms and 120ms respectively, while the alternans and 2:1 block of the His bundle happened at 120ms and 110ms respectively.

## Discussion

The main findings from this study are as follows: 1) The His bundle can be captured at shorter cycle lengths than the WM. 2) The APD of the His bundle is shorter than the APD of the WM. 3) Alternans develop in the His bundle at short cycle lengths with His bundle pacing when 2:1 conduction block occurs in the WM.

As mentioned previously, the PS plays a significant role as a driver of the rapid activation rates of VF, particularly after the first 2–3 minutes of VF.[1–3] An excitable gap exists in the WM during VF[9] and the results of our study demonstrate that the APD of the His bundle is shorter than that of the WM across a broad range of CLs. This is consistent with the hypothesis that an excitable gap may exist in the His bundle during VF. For fibrillatory activations to move through a large portion of the conduction system and to spread to the other side of the heart, an excitable gap in the His bundle would be necessary. Our previous studies have shown that during VF, a synchronized pattern of excitation on the endocardium emerges that is similar to the spread of activation through the conduction system to the endocardial left ventricular working myocardium during sinus rhythm.[10] A possible explanation of the same pattern of endocardial activation occurring in sinus rhythm and during VF would be that activation is being triggered from the right ventricular conduction system or superior to the His bundle, and then activation spread through the LV conduction system and to the LV myocardium. For this to be possible, activation would likely have to had passed through the His bundle. The present study demonstrates that the His bundle characteristics are consistent with the His bundle having a shorter APD and refractory period than the working myocardium so that activation could pass through the His bundle at fibrillation-like cycle lengths.

Alternans has been shown to be related to conduction block and potentially to arrhythmia onset. Because of the slow propagation and long refractory period of the atrioventricular node (AVN), alternans in the AVN and phenomenon such as Wenchebach conduction through the AVN node are well documented.[11, 12] Our results showed that the ventricular alternans developed in longer CL than His bundle alternans, which suggests that the ventricle lost 1:1 capture in longer CL than the His bundle. In other words, when the His bundle had alternans, 2:1 block occurred in the WM. Cardiac elelctrophysiology modeling and experimental studies show that the combination of the voltage and timing dependence of the inward rectifier currents (I_Kr_) lead to alternans onset when the cycle length is approaching the refractory period of the tissue.[13] Similar to results presented in the current study, other investigators have previously reported alternans in Purkinje and myocardial tissue during dynamic restitution pacing protocols.[14, 15]. While alternans in working myocardium seem to be highly affected by Ca++ levels and handling,[16] Purkinje alternans are more independent of Ca++ and appear to be driven by factors driving APD (alternans in previous diastolic interval). Further studies will be required to determine the role of Ca++ handling in alternans development in the His bundle.[15] For the shorter AP in the His bundle, less current (the source) was generated, which may be not have been sufficient to bring the adjacent repolarized tissue (the sink) to its activation threshold, and propagation may fail as the source-sink mismatch is too large[17–21]. However, there is another viewpoint that the alternans might be due to electrotonic coupling with a nearby area showing 2:1 block, resulting in ‘‘secondary” alternans. Strong electrical communication via gap junction with other cells may cause a smoothing of the Ca2+ signal over many cells, preventing alternans (voltage-Ca2+ feedback)[22, 23]. There are also some studies that demonstrated that partial gap junction inhibition did have a strong effect on Ca2+ transient alternans, significantly increasing the occurrence and intensity.[22, 24] From our results, it was difficult to determine definitively the relationship between the His bundle alternans and ventricular 2:1 block. Thus, further studies are needed to explore the mechanism of the His alternans.

### Study limitation

As the His bundle area of rabbit heart is small and superficial, recording successive APs from the His bundle when pacing is a challenging task. Any motion of the heart or the perfusate had a great effect on the recording. We had to try different sites of the His bundle area to obtain stable recordings. Thus, the APs recordings from individual animals might be not from the same section of the His bundle (i.e. proximal or distal His bundle). Also, in order to reduce the motion of the heart, blebbistatin was used in our study, which may have an effect on action potential characteristics.[25]

Another limitation is that recordings of WM were only made from the high RV septal endocardium, which may not represent the restitution properties in the whole ventricle. As some studies have reported that electrophysiological characteristics of epicardium, endocardium and midmyocardium are different[26, 27], further studies are needed to compare the electrophysiological differences between the His bundle and the epicardium, midmyocardium, apex and base myocardial myocytes, AV node, Bundle branches, and Purkinje fibers.

In addition, we used AUCs for comparing the relationship between APS and CL between the His bundle and WM. Although the AUC measure showed that the relationship between APD and CL is different between the His bundle and WM, it did not indicate how exactly the shape of the curves were different. Using AUC as the measure for the relationship between APD and CL is due to the lack of reference that can establish the functional relationship (linear or non-linear) between APD and CL. This underscores the need of further experiments for establishing the mathematical function for the relationship between APD and CL.

## Conclusion

The APD of the His bundle was significantly shorter than that of the underlying WM, which is supportive of short wavefronts propagating through the through the His bundle to other portions of the specialized conduction system at both short and long CL. Alternans were observed in the His bundle APD at short cycle lengths when conduction block occurred prior to wavefronts arriving at the WM underlying the His bundle. Further studies are needed to identify the location of conduction block along the conduction system distal to the His bundle or in the working myocardium at short CLs.

## References

[1] Tabereaux PB, Walcott GP, Rogers JM, Kim J, Dosdall DJ, Robertson PG, et al Activation patterns of Purkinje fibers during long-duration ventricular fibrillation in an isolated canine heart model. Circulation. 2007;116(10):1113–9. Epub 2007/08/19. doi: CIRCULATIONAHA.107.699264 [pii] doi: 10.1161/CIRCULATIONAHA.107.699264 . 17698730 10.1161/CIRCULATIONAHA.107.699264

[2] Dosdall DJ, Tabereaux PB, Kim JJ, Walcott GP, Rogers JM, Killingsworth CR, et al Chemical ablation of the Purkinje system causes early termination and activation rate slowing of long-duration ventricular fibrillation in dogs. American journal of physiology Heart and circulatory physiology. 2008;295(2):H883–9. doi: 10.1152/ajpheart.00466.2008 ; PubMed Central PMCID: PMC2519198. 18586887 10.1152/ajpheart.00466.2008 PMC2519198

[3] Huang J, Dosdall DJ, Cheng KA, Li L, Rogers JM, Ideker RE. The importance of Purkinje activation in long duration ventricular fibrillation. Journal of the American Heart Association. 2014;3(1):e000495 doi: 10.1161/JAHA.113.000495 ; PubMed Central PMCID: PMC3959715. 24584738 10.1161/JAHA.113.000495 PMC3959715

[4] Robinson RB, Boyden PA, Hoffman BF, Hewett KW. Electrical restitution process in dispersed canine cardiac Purkinje and ventricular cells. Am J Physiol. 1987;253(5 Pt 2):H1018–25. Epub 1987/11/01. . 3688246 10.1152/ajpheart.1987.253.5.H1018

[5] Angel N, Li L, Dosdall DJ. His Bundle Activates Faster than Ventricular Myocardium during Prolonged Ventricular Fibrillation. PLoS One. 2014;9(7):e101666 Epub 2014/07/19. doi: 10.1371/journal.pone.0101666 . 25036418 10.1371/journal.pone.0101666 PMC4103805

[6] Guide for the Care and Use of Laboratory Animals. The National Academies Collection: Reports funded by National Institutes of Health. 8th ed. Washington (DC)2011.

[7] Fedorov VV, Lozinsky IT, Sosunov EA, Anyukhovsky EP, Rosen MR, Balke CW, et al Application of blebbistatin as an excitation-contraction uncoupler for electrophysiologic study of rat and rabbit hearts. Heart rhythm: the official journal of the Heart Rhythm Society. 2007;4(5):619–26. doi: 10.1016/j.hrthm.2006.12.047 . 17467631 10.1016/j.hrthm.2006.12.047

[8] Huang J, Zhou X, Smith WM, Ideker RE. Restitution properties during ventricular fibrillation in the in situ swine heart. Circulation. 2004;110(20):3161–7. Epub 2004/11/10. doi: 10.1161/01.CIR.0000147618.93579.56 . 15533856 10.1161/01.CIR.0000147618.93579.56

[9] KenKnight BH, Bayly PV, Gerstle RJ, Rollins DL, Wolf PD, Smith WM, et al Regional capture of fibrillating ventricular myocardium. Evidence of an excitable gap. Circulation research. 1995;77(4):849–55. . 7554132 10.1161/01.res.77.4.849

[10] Li L, Zheng X, Dosdall DJ, Huang J, Pogwizd SM, Ideker RE. Long-duration ventricular fibrillation exhibits 2 distinct organized states. Circ Arrhythm Electrophysiol. 2013;6(6):1192–9. Epub 2013/11/19. doi: 10.1161/CIRCEP.113.000459 . 24243784 10.1161/CIRCEP.113.000459 PMC3982786

[11] Zhang Y. His electrogram alternans (Zhang's phenomenon) and a new model of dual pathway atrioventricular node conduction. Journal of interventional cardiac electrophysiology: an international journal of arrhythmias and pacing. 2016;45(1):19–28. doi: 10.1007/s10840-015-0079-0 . 26614299 10.1007/s10840-015-0079-0

[12] Zhang Y, Mazgalev TN. AV nodal dual pathway electrophysiology and Wenckebach periodicity. Journal of cardiovascular electrophysiology. 2011;22(11):1256–62. doi: 10.1111/j.1540-8167.2011.02068.x . 21489031 10.1111/j.1540-8167.2011.02068.x

[13] Hua F, Gilmour RF Jr. Contribution of IKr to rate-dependent action potential dynamics in canine endocardium. Circ Res. 2004;94(6):810–9. doi: 10.1161/01.RES.0000121102.24277.89 . 14963001 10.1161/01.RES.0000121102.24277.89

[14] Koller ML, Riccio ML, Gilmour RF Jr. Dynamic restitution of action potential duration during electrical alternans and ventricular fibrillation. Am J Physiol. 1998;275(5 Pt 2):H1635–42. . 9815071 10.1152/ajpheart.1998.275.5.H1635

[15] Saitoh H, Bailey JC, Surawicz B. Action potential duration alternans in dog Purkinje and ventricular muscle fibers. Further evidence in support of two different mechanisms. Circulation. 1989;80(5):1421–31. . 2553299 10.1161/01.cir.80.5.1421

[16] Goldhaber JI, Xie LH, Duong T, Motter C, Khuu K, Weiss JN. Action potential duration restitution and alternans in rabbit ventricular myocytes: the key role of intracellular calcium cycling. Circ Res. 2005;96(4):459–66. doi: 10.1161/01.RES.0000156891.66893.83 . 15662034 10.1161/01.RES.0000156891.66893.83

[17] Joyner RW, Sugiura H, Tan RC. Unidirectional block between isolated rabbit ventricular cells coupled by a variable resistance. Biophysical journal. 1991;60(5):1038–45. doi: 10.1016/S0006-3495(91)82141-5 ; PubMed Central PMCID: PMC1260161. 1760503 10.1016/S0006-3495(91)82141-5 PMC1260161

[18] Shaw RM, Rudy Y. Ionic mechanisms of propagation in cardiac tissue. Roles of the sodium and L-type calcium currents during reduced excitability and decreased gap junction coupling. Circulation research. 1997;81(5):727–41. . 9351447 10.1161/01.res.81.5.727

[19] Spach MS, Boineau JP. Microfibrosis produces electrical load variations due to loss of side-to-side cell connections: a major mechanism of structural heart disease arrhythmias. Pacing and clinical electrophysiology: PACE. 1997;20(2 Pt 2):397–413. . 9058844 10.1111/j.1540-8159.1997.tb06199.x

[20] Rohr S, Kucera JP, Fast VG, Kleber AG. Paradoxical improvement of impulse conduction in cardiac tissue by partial cellular uncoupling. Science (New York, NY). 1997;275(5301):841–4. . 9012353 10.1126/science.275.5301.841

[21] Plotnikov AN, Shlapakova I, Szabolcs MJ, Danilo P Jr., Lorell BH, Potapova IA, et al Xenografted adult human mesenchymal stem cells provide a platform for sustained biological pacemaker function in canine heart. Circulation. 2007;116(7):706–13. doi: 10.1161/CIRCULATIONAHA.107.703231 . 17646577 10.1161/CIRCULATIONAHA.107.703231

[22] Hammer KP, Ljubojevic S, Ripplinger CM, Pieske BM, Bers DM. Cardiac myocyte alternans in intact heart: Influence of cell-cell coupling and beta-adrenergic stimulation. Journal of molecular and cellular cardiology. 2015;84:1–9. doi: 10.1016/j.yjmcc.2015.03.012 ; PubMed Central PMCID: PMC4500534. 25828762 10.1016/j.yjmcc.2015.03.012 PMC4500534

[23] Sato D, Bers DM, Shiferaw Y. Formation of spatially discordant alternans due to fluctuations and diffusion of calcium. PloS one. 2013;8(12):e85365 doi: 10.1371/journal.pone.0085365 ; PubMed Central PMCID: PMC3877395. 24392005 10.1371/journal.pone.0085365 PMC3877395

[24] Jia Z, Bien H, Shiferaw Y, Entcheva E. Cardiac cellular coupling and the spread of early instabilities in intracellular Ca2+. Biophysical journal. 2012;102(6):1294–302. doi: 10.1016/j.bpj.2012.02.034 ; PubMed Central PMCID: PMC3309286. 22455912 10.1016/j.bpj.2012.02.034 PMC3309286

[25] Brack KE, Narang R, Winter J, Ng GA. The mechanical uncoupler blebbistatin is associated with significant electrophysiological effects in the isolated rabbit heart. Experimental physiology. 2013;98(5):1009–27. doi: 10.1113/expphysiol.2012.069369 ; PubMed Central PMCID: PMC3734628. 23291912 10.1113/expphysiol.2012.069369 PMC3734628

[26] Myles RC, Bernus O, Burton FL, Cobbe SM, Smith GL. Effect of activation sequence on transmural patterns of repolarization and action potential duration in rabbit ventricular myocardium. American journal of physiology Heart and circulatory physiology. 2010;299(6):H1812–22. doi: 10.1152/ajpheart.00518.2010 ; PubMed Central PMCID: PMC3006295. 20889843 10.1152/ajpheart.00518.2010 PMC3006295

[27] Anyukhovsky EP, Sosunov EA, Rosen MR. Regional differences in electrophysiological properties of epicardium, midmyocardium, and endocardium. In vitro and in vivo correlations. Circulation. 1996;94(8):1981–8. . 8873677 10.1161/01.cir.94.8.1981

